# Zoning has little impact on the seasonal diel activity and distribution patterns of wild boar (*Sus scrofa*) in an UNESCO Biosphere Reserve

**DOI:** 10.1002/ece3.8347

**Published:** 2021-11-23

**Authors:** Henrik Reinke, Hannes J. König, Oliver Keuling, Tobias Kuemmerle, Christian Kiffner

**Affiliations:** ^1^ Junior Research Group Human‐Wildlife Conflict & Coexistence Leibniz Centre for Agricultural Landscape Research (ZALF) Müncheberg Germany; ^2^ Geography Department Humboldt‐Universität zu Berlin Berlin Germany; ^3^ Institute for Terrestrial and Aquatic Wildlife Research (ITAW) University of Veterinary Medicine Hannover Hannover Germany; ^4^ Integrative Research Institute on Transformations of Human‐Environment Systems IRI THESys Berlin Germany

**Keywords:** activity patterns, circadian rhythm, human–wildlife conflict, protected area management, seasonality, wildlife management

## Abstract

Understanding the spatio‐temporal distribution of ungulates is important for effective wildlife management, particularly for economically and ecologically important species such as wild boar (*Sus scrofa*). Wild boars are generally considered to exhibit substantial behavioral flexibility, but it is unclear how their behavior varies across different conservation management regimes and levels of human pressure. To analyze if and how wild boars adjust their space use or their temporal niche, we surveyed wild boars across the core and buffer zones (collectively referred to as the conservation zone) and the transition zone of a biosphere reserve. These zones represent low and high levels of human pressure, respectively. Specifically, we employed a network of 53 camera traps distributed in the Schaalsee UNESCO Biosphere Reserve over a 14‐month period (19,062 trap nights) and estimated circadian activity patterns, diel activity levels, and occupancy of wild boars in both zones. To account for differences in environmental conditions and day length, we estimated these parameters separately for seven 2‐month periods. Our results showed that the wild boars were primarily nocturnal, with diurnal activity occurring dominantly during the summer months. The diel activity patterns in the two zones were very similar overall, although the wild boars were slightly less active in the transition zone than in the conservation zone. Diel activity levels also varied seasonally, ranging from 7.5 to 11.0 h day^−1^, and scaled positively with the length of the night (*R* ^2^ = 0.66–0.67). Seasonal occupancy estimates were exceptionally high (point estimates ranged from 0.65 to 0.99) and similar across zones, suggesting that the wild boars used most of the biosphere reserve. Overall, this result suggests that different conservation management regimes (in this case, the zoning of a biosphere reserve) have little impact on wild boar behavior. This finding is relevant for wildlife management in protected areas where possibly high wild boar densities could interfere with conservation goals within these areas and those of agricultural land use in their vicinity.

## Graphical abstract

In this study, we analyzed whether wild boars adjust their space use or their temporal niche according to spatial differences in management within a biosphere reserve. We employed a network of 53 camera traps distributed in the Schaalsee UNESCO Biosphere Reserve over a 14‐month period (19,062 trap nights) and estimated the circadian activity patterns, activity levels, and occupancy of wild boars in the conservation and transition zones; these zones represent low and high levels of human pressure, respectively. Wild boars were primarily nocturnal, diel activity levels were positively correlated with the length of the night and did not differ markedly between zones; occupancy estimates were high and were similar in both zones . Overall, these results suggests that different conservation management regimes (in this case, the zoning of a biosphere reserve) have little impact on wild boar behavior.

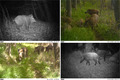

## 1 INTRODUCTION

Land use and other human pressures have substantial, yet complex consequences for wildlife populations (Tucker et al., 2020). Many large mammal species are in decline due to anthropogenic impacts (Bar‐On et al., 2018; Craigie et al., 2010) and are subject to species filtering in human‐dominated landscapes (Brashares, 2010; Riggio et al., 2018). In contrast, some species can persist or even thrive in human‐dominated landscapes (Tucker et al., 2020). This is exemplified by the rebounding of the populations of European large mammals, especially of many European ungulates (Apollonio et al., 2010; Chapron et al., 2014). This is particularly the case for roe deer (*Capreolus capreolus*), red deer (*Cervus elaphus*), and wild boar (*Sus scrofa*), populations of which have increased markedly during recent decades (Carpio et al., 2020; Massei et al., 2015; Milner et al., 2006). What remains unclear in this context, however, is how space use of these ungulates has changed and whether it varies in relation to varying levels of human pressure.

The population and distributional range of wild boar have markedly increased in recent times, with concomitant increases in the species' impact on other organisms and human livelihoods (Barrios‐Garcia & Ballari, 2012). The foraging behavior of wild boar (i.e., rooting: overturning the soil in search of food) leads to substantial crop and grassland damage (Cocca et al., 2007; Herrero et al., 2006; Schley et al., 2008; Schley & Roper, 2003). Furthermore, rooting can have negative effects on plant cover, diversity, and regeneration (Barrios‐Garcia & Ballari, 2012), and wild boar behavior can therefore be in conflict with conservation goals. For example, the foraging behavior of wild boar can compromise the reproductive success of ground‐breeding bird species (Carpio et al., 2014; Oja et al., 2017), threaten reptiles (Graitson et al., 2019), and negatively impact plant biodiversity (Hone, 2002). The recent increase in the wild boar population size has primarily been attributed to higher food availability due to agricultural intensification as well as milder winters due to climate change (Barrios‐Garcia & Ballari, 2012; Massei et al., 2015; Vetter et al., 2015, 2020). Attempts to control wild boar populations through recreational hunting alone are typically considered inefficient and ineffective (Keuling et al., 2013, 2016), and the relative impact of hunting on wild boar mortality has recently decreased (Massei et al., 2015). Nevertheless, hunting remains the main cause of wild boar mortality in Central Europe (Keuling et al., 2013).

**FIGURE 1:**
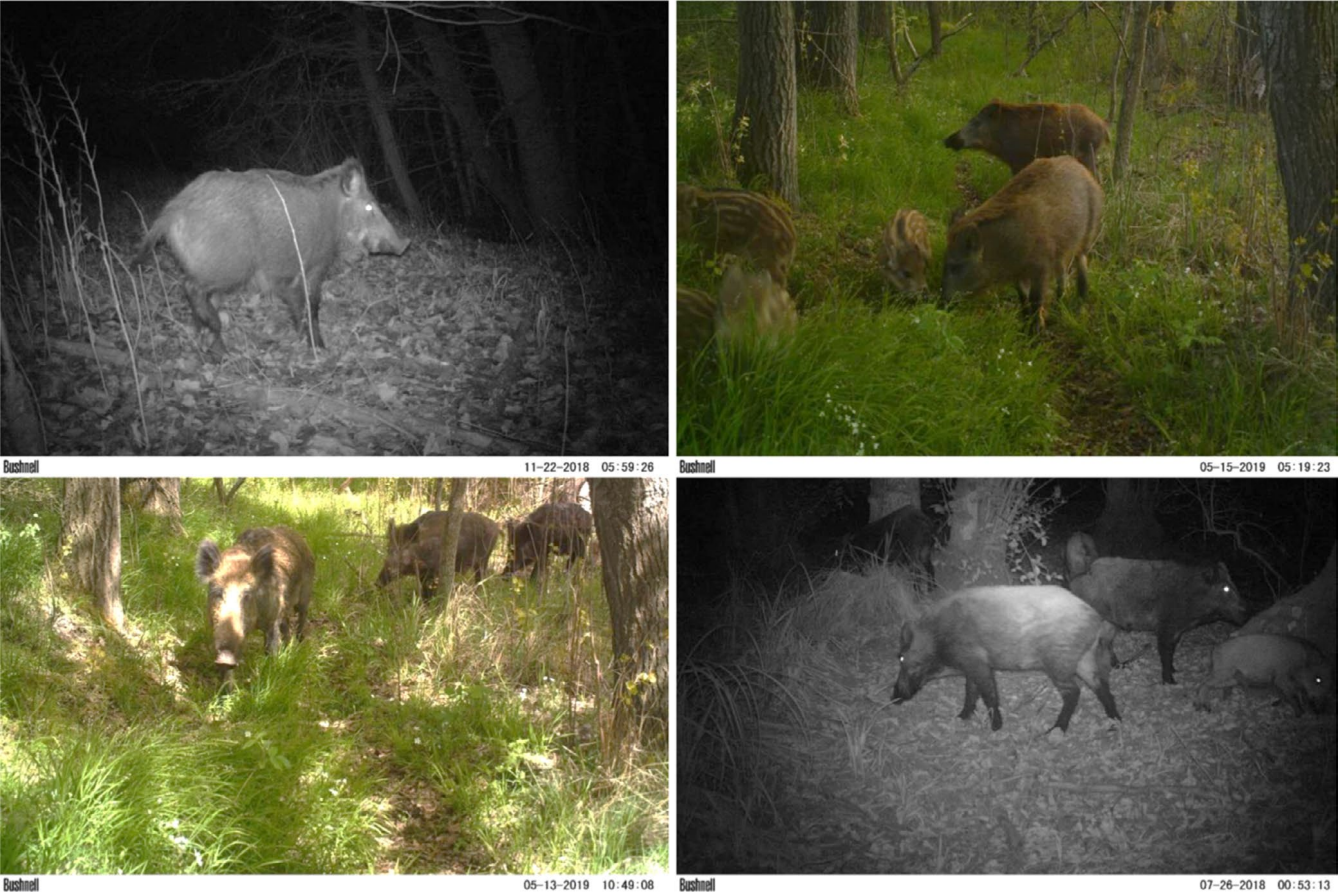
Exemplary camera trap pictures of wild boar (*Sus scrofa*) in the Schaalsee Biosphere Reserve

The success of wild boar populations in human‐dominated landscapes can in part be attributed to the substantial plasticity of wild boar behavior (Lemel et al., 2003; Podgorski et al., 2013). Wild boars are habitat generalists and can survive well in a wide variety of habitats, including agricultural fields, which are primarily used during the growing season (Amendolia et al., 2019; Johann et al., 2020; Morelle & Lejeune, 2015; Morelle et al., 2015; Thurfjell et al., 2009). Wild boars can adjust the daily temporal patterns of their behavior (Figure 1) and become almost exclusively nocturnal in areas that are subject to hunting or greater human activity (Brivio et al., 2017; van Doormaal et al., 2015; Johann, Handschuh, Linderoth, Dormann, et al., 2020; Ohashi et al., 2013; Russo et al., 1997). Additionally, seasonal changes (e.g., a decrease in resource availability and hiding cover) can mediate their spatial and temporal use and avoidance of human‐impacted landscapes. For example, Viola et al. (2021) registered significant increases in wild boar abundance from early summer to autumn and hypothesized that these temporal shifts in the home range were related to the loss of hiding cover after the agricultural harvest period. Likewise, urban and rural wild boars perceive humans differently, most likely due to their differential habituation to humans (Stillfried et al., 2017). Nevertheless, urban wild boars still strongly prefer natural landscapes (Stillfried et al., 2017) and the availability of (semi‐)natural hiding places appears to be crucial for wild boar to cope with stressful situations (Rutten et al., 2020). This effect is also indicated by increased crop damage to agricultural fields in the vicinity of forests (Thurfjell et al., 2009), especially those where hunting is restricted or banned (Amici et al., 2012). In heavily hunted wild boar populations, both spatial and temporal behavioral adjustments in response to differences in human activity have been observed (e.g. Johann, Handschuh, Linderoth, Dormann, et al., 2020; Johann, Handschuh, Linderoth, Heurich, et al., 2020; Keuling et al., 2008b; Massei et al., 2015). A better understanding the wide variability of wild boar behavior in human‐dominated landscapes is thus particularly relevant for conservation planning in protected areas.

UNESCO biosphere reserves represent interesting quasi‐experimental settings to assess the effects of different human pressures on the behavior of wildlife and wild boar specifically. Biosphere reserves are designed to integrate the management of natural resources by combining conservation and human development through regional zoning. The zonal structure of biosphere reserves can serve as a useful proxy for a certain level of anthropogenic disturbance because each zone represents a defined intensity of human impact. The strictly protected *core zone* is characterized by a minimum of human intervention, and access is restricted to research, monitoring, or educational purposes only. It is surrounded or adjoined by the *buffer zone* (high protection level) with the objective of conserving species‐rich cultural landscapes. The core and buffer zone (which are considered together in this study as the “conservation zone”) are in turn surrounded by the *transition zone*, which has low or no protection status and consists mostly of agricultural land and settlements. The goal of the transition zone is to achieve sustainable development in the wider area (UNESCO, 2017). Given these different protection levels, wild boar in the conservation zone should be less exposed to human disturbance and predation risk than those in the transition zone. As wildlife species can change their diel activity patterns toward greater nocturnal activity when subject to human disturbance (Gaynor et al., 2018), we would expect that (1) wild boar in the conservation zone exhibit more diurnal activity than wild boar in the transition zone. In addition, we would expect that wild boar primarily used less disturbed areas and thus hypothesized that (2) the occupancy of wild boar is greater in the conservation zone than in the transition zone. Finally, we would expect that (3) space use and diel activity patterns in wild boar are mediated by seasonal effects, as wild boar may adjust their space use according to variations in seasonal resource availability. For example, between May and October, wild boar often use agricultural fields until harvest (Keuling et al., 2009), and the home ranges of female wild boar may be greater during the summer months than during the winter months (Keuling et al., 2008a). Thus, we would expect a greater occupancy of wild boar in the transition zone during the summer months than during the winter months. In areas distant from the equator, where the day length varies (Vazquez et al., 2019), seasonality could also affect the timing of wild boar activity (Keuling et al., 2008b). If wild boar are primarily nocturnal and if sunset and sunrise are crucial triggers of wild boar activity (Hill et al., 2003; Lemel et al., 2003), we would expect that wild boar activity during the summer months would start later in the evening and end earlier in the morning than that in the winter months and that diel activity levels (i.e., the amount of time that wild boar are active) would be positively correlated with the length of the night. To test these specific predictions, we surveyed wild boar along a systematic network of camera traps distributed in the conservation and transition zones of the Schaalsee Biosphere Reserve over a 14‐month period.

## 2 MATERIAL AND METHODS

### 2.1 Study area

**FIGURE 2:**
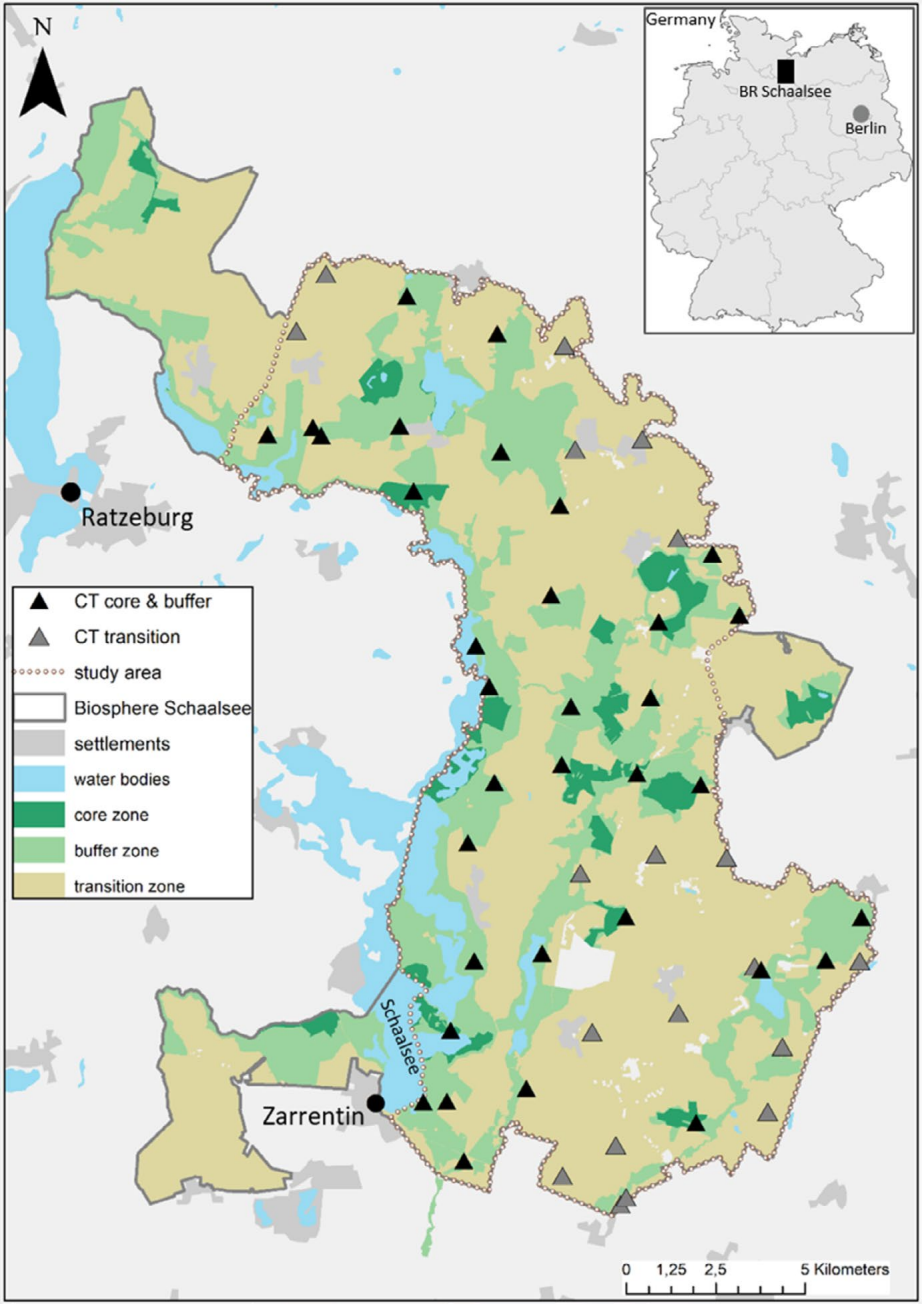
Location of camera trap stations, management zones, and key landscape features of the study area in the Schaalsee Biosphere Reserve. The inset at the top right indicates the location of the study area within Germany

The Schaalsee UNESCO Biosphere Reserve is located in northern Germany in the center of the conurbations of Hamburg, Lübeck, and Schwerin (central coordinates: 53°39′09″N, 10°59′25″E). It covers an area of approximately 310 km^2^ (expansion from north to south: ~32 km; from east to west: ~12 km) along the eastern shore of the Lake Schaalsee (Figure 2). Schaalsee was a natural part of the closed inner German border between 1961 and 1989. This situation supported undisturbed natural development and thereby created the basis for its designation as a biosphere reserve. The reserve is approximately 49% arable land, 21% grassland, 19% forest, 8% lakes and small water bodies, and 3% settlements and roads. The main biome type is a Baltic beech forest. The biosphere reserve comprises 18 nature conservation areas covering approximately 25% of its total area. Like all biosphere reserves, it is divided into three different zones: (1) the *core zone* (17.6 km^2^; ~6% of the total area), (2) the *buffer zone* (92.4 km^2^; ~29%), and the (3) *transition zone* (195.7 km^2^; ~65%).

The region is influenced by both sub‐Atlantic and subcontinental climates, as it is located in the intermediate area between these two climate zones. During the study period (July 2018–August 2019), the mean air temperature was 12°C. The lowest air temperature was reached in January 2019, at −8°C, and the highest was reached in July 2018, at 36°C. The average precipitation during the camera trapping session was 41 mm per month (mean variance of the daily precipitation: 16.2). The lowest mean precipitation levels during the study period were recorded in November 2018 (11.9 mm) and April 2019 (13.6 mm), while March 2019 (75.6 mm) and June 2019 (119.6 mm) were the rainiest months (information derived from the reserve's weather station in Zarrentin).

Hunting in the Schaalsee Biosphere Reserve is carried out by private hunters and professional foresters. The two administrative districts of the region comprise approximately 80 hunting districts. The mean annual hunting bag for wild boar in these hunting districts between 2012 and 2018 was about 1,280 individuals, with a rising trend over time. There are no closed seasons for wild boar hunting, aside from a general hunting ban on mother sows. Single hunts from blinds or by stalking take place year‐round. The use of bait sites to facilitate hunting is common. Additionally, driven hunts are performed between October and January. In the core zone, hunting is allowed only to avoid wildlife damages to the adjacent agricultural fields or for animal health purposes. Aside from these situations, there is a fundamental ban on human interference and thus also on the management of game ungulates in core zone areas. In the buffer and transition zones, there are no special restrictions in terms of hunting. The difference between the buffer and transition zones is thus mostly characterized by the intensity of agriculture.

### 2.2 Camera trap survey

We established our study area as a contiguous area of approximately 200 km^2^ by clipping approximately 110 km^2^ of the total reserve (see the dotted line in Figure 2). We then placed a raster layer of 4 × 4 km^2^ cells over the study area and marked one potential location for camera placement within each cell on the basis of aerial imagery. For logistical and data protection reasons, we restricted the camera locations to state forest areas or areas that are owned by the reserve administration, the World Wildlife Fund (WWF) or the Schaalsee‐Landschaft Communal Association (*Zweckverband*). In the field, we navigated to the predetermined camera location and scanned the surrounding area for wildlife trails, paths, or tracks. We attached camera traps (Bushnell TrophyCam and TrophyCam HD) to either a tree or a wooden pole (positioned with a metal ground sleeve) at a height of between 0.5 m and 0.9 m and fixed all cameras on stands, which allowed the camera trap's orientation and inclination to be adjusted. This facilitated the positioning of the cameras, especially on hilly terrain, to ensure that an individual of the target species would be fully visible in the photographs at the optimal detection range of the devices at approximately 10 m. We placed the camera traps in June 2018, and they were operational until we recovered them in August 2019 (unless cameras were malfunctioning or stolen). We visited them once per month for maintenance and to exchange memory cards. Four Bushnell camera traps were stolen during the study period and were replaced by Stealthcam devices (model GX45NG). In total, we set up 53 camera trap stations, of which 19 stations were established in the transition zone and 34 in the conservation zone.

We processed, organized, and tagged all images using the open‐source software CAMELOT version 1.6.2 (Hendry & Mann, 2018). The tags included the species as well as the number of individuals, sex, and age class if recognizable. In line with other camera trap research, we counted two observations of the same species as independent if they were at least 30 min apart (Briceno‐Mendez et al., 2016; Havmøller et al., 2020). For the statistical analyses, we grouped the wild boar events into seven seasonal clusters. Each cluster comprised 2 months of data and represented a time period of similar day length and vegetation phenology and contained a sufficient number of detections to model activity patterns as well as capture histories to model occupancy. The seasonal clusters were defined as follows: late summer 2018 (LS2018): July–August; fall 2018 (F2018): September–October; winter 2018 (W2018): November–December; winter 2019 (W2019): January–February; spring 2019 (S2019): March–April; early summer 2019 (ES2019): May–June; and late summer 2019 (LS2019): July–August. For each bimonthly period, we obtained the night length of the last day of the first month (sunrise‐and‐sunset.com, 2020). We considered the images from the camera traps from the conservation zone (i.e., the combined core and buffer zones) together because we had few cameras in the core zone and because the two zones do not differ much with respect to human impacts that could affect wild boar behavior.

### 2.3 Statistical analysis

To analyze and illustrate wild boar diel activity patterns, we used the *activity* (Rowcliffe, 2019; Rowcliffe et al., 2014) and *overlap* (Ridout & Linkie, 2009) R packages (R Core Team, 2019). Based on the time‐stamped photographs, we modeled the circadian rhythms of wild boar for each bimonthly period and management zone. We conducted pairwise comparisons (i.e., time‐matched comparisons between the conservation and transition zones) of the diel activity distributions (i.e., assessing the overlap and shape of the distributions) and of the wild boar diel activity levels (i.e., assessing the proportion of daily activity) using the *compareCkern* and *compareAct* functions, respectively. For all comparisons, we used 1000 bootstrap replicates. To broadly describe how much of the wild boar activity occurred during the daytime (defined here as the time after sunrise and before sunset) and the nighttime (defined as the time after sunset and before sunrise), we calculated the proportions of daytime and nighttime camera events for each season and management zone. To further analyze whether diel activity levels were associated with night length, we plotted the duration of active time day^−1^ (calculated by multiplying the activity level by 24 h) against the night length (defined as the time period between sunset and sunrise) during each bimonthly period and fitted separate linear regressions for these relationships in both zones of the reserve.

To assess the broad‐scale distribution patterns of wild boar in the study area, we fitted single‐season occupancy models (MacKenzie et al., 2017) for each time period in the R package *unmarked* (Fiske & Chandler, 2011). For the entire survey period, we created capture histories of wild boar at each station (presence = 1, absence = 0) based on the calendar weeks. For each bimonthly period, we matched the calendar weeks as closely as possible, generally resulting in 9 weeks per bimonthly period (except for W2019 = 8 weeks and LS 2019 = 7 weeks), and used these capture histories for occupancy modeling. When cameras were stolen or malfunctioning for more than three days per calendar week, we assigned “NA” to the corresponding cell and week.

Because sampling effort differed across zones, and as we were interested in broad‐scale differences in the distribution of wild boar, we evaluated four candidate occupancy models: (1) an intercept only model where detection probability (*p*) and occupancy (*psi*/ψ) are constant, (2) one model with constant *p* and *psi* conditional on the zone of the biosphere reserve, (3) one candidate model with constant *psi* and *p* conditional on the zone of the biosphere reserve, and (4) the most complex model where *psi* and *p* are conditional on the zone of the biosphere reserve. To account for multiple models receiving similar support, we used model‐averaging to aggregate models that performed equally (ΔAICc < 2) using the *AICcmodavg* package in R (Mazerolle, 2020). We back‐transformed the model‐averaged occupancy and detection probability estimates using the *predict* function and plotted these bimonthly estimates.

## 3 RESULTS

Our 14‐month‐long camera trap survey yielded a total of 4,017 wild boar events (core & buffer zone: 2,903; transition zone: 1,114) from an effort of 19,062 camera trap days (core & buffer zone: 12,154; transition zone: 6,872). Based on the camera trapping rates, the species with the highest relative abundance was roe deer (43% of all camera trap events), followed by wild boar (25%). Red deer (1.1%) and fallow deer (0.4%) were considerably less abundant. Invasive raccoons (*Procyon lotor*) ranked third, at 10% of all observations and thus showed a higher relative abundance than autochthonous red foxes (*Vulpes vulpes*), at 7% of all observations.

**FIGURE 3:**
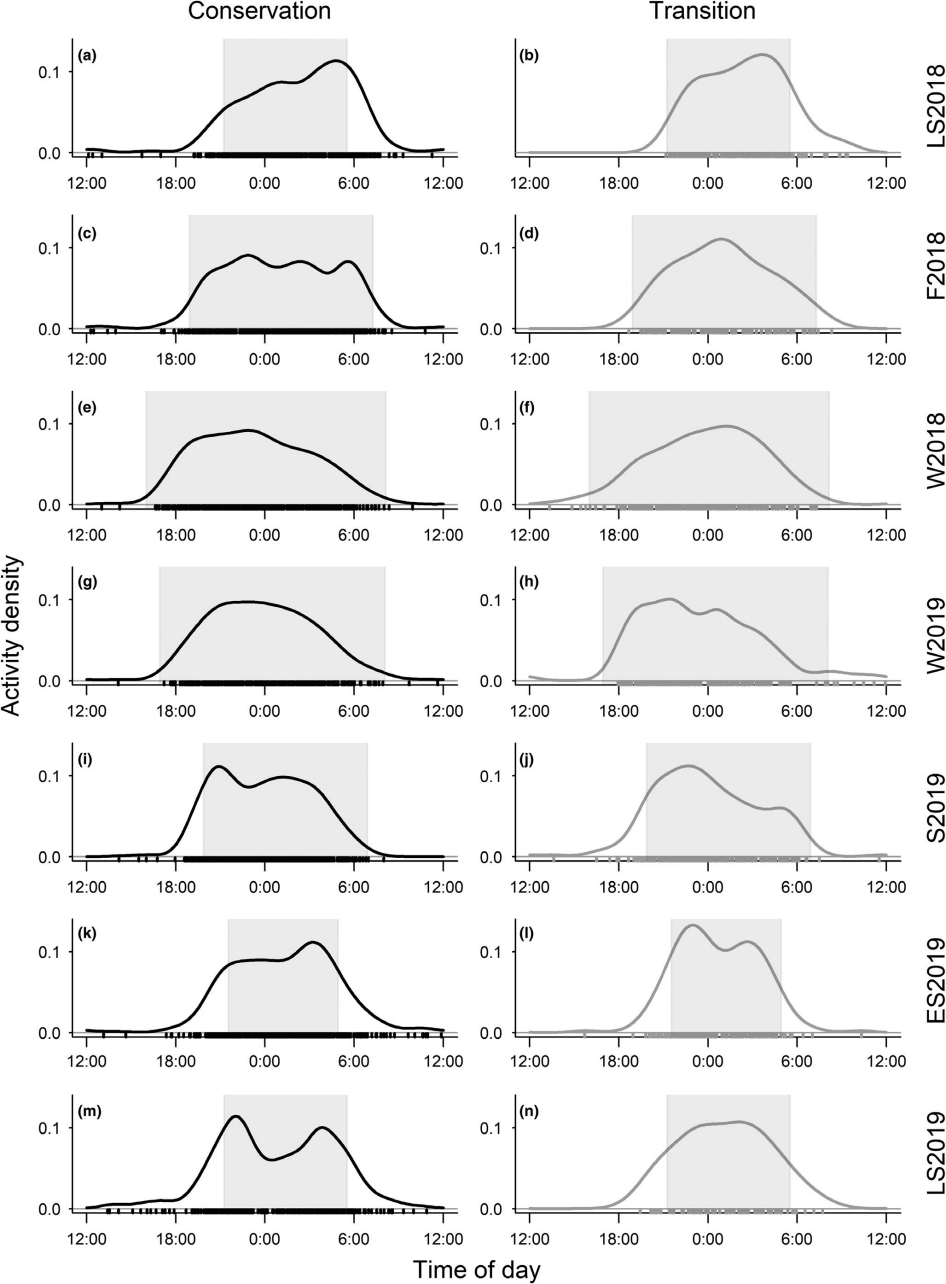
Bimonthly activity patterns of wild boar (*Sus scrofa*) in the conservation (a, c, e, g, I, k, m) and transition (b, d, f, h, j, l, n) zones of the Schaalsee Biosphere Reserve, Germany. LS2018: July–August 2018 (a, b); F2018: September–October 2018 (c, d); W2018: November–December 2018 (e, f); W2019: January–February 2019 (g, h); S2019: March–April 2019 (i, j); ES2019: May‐June 2019 (k, l); LS2019: July–August 2019 (m, n). The gray shaded areas represent activities at nighttime, defined as the time between sunset and sunrise

**FIGURE 4:**
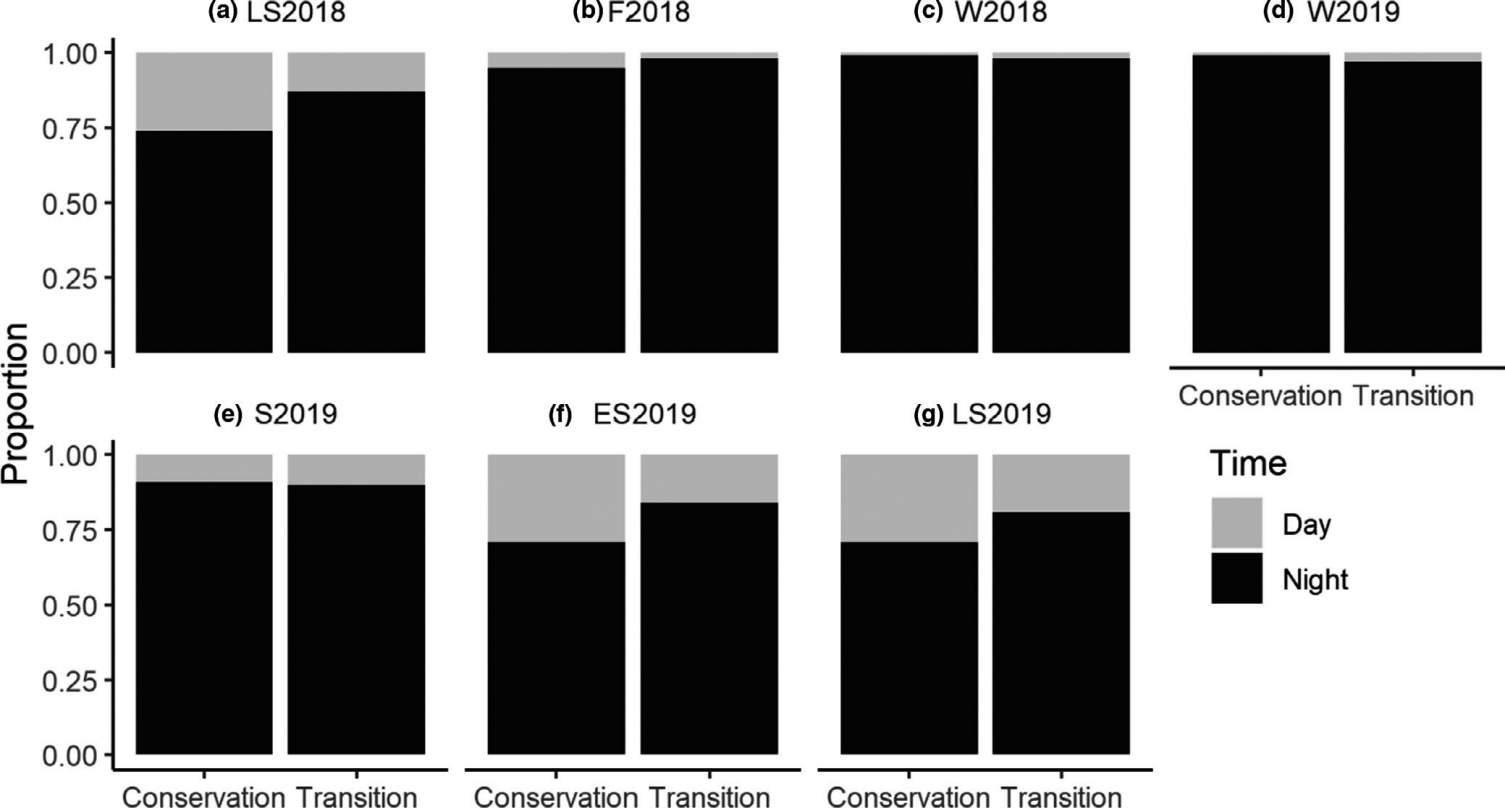
Proportion of nighttime and daytime camera trap events for wild boar in the conservation and transition zones of the Schaalsee Biosphere Reserve during bimonthly (LS2018: July–August 2018; F2018: September–October 2018; W2018: November–December 2018; W2019: January–February 2019; S2019: March–April 2019; ES2019: May–June 2019; LS2019: July–August 2019) time periods

**FIGURE 5:**
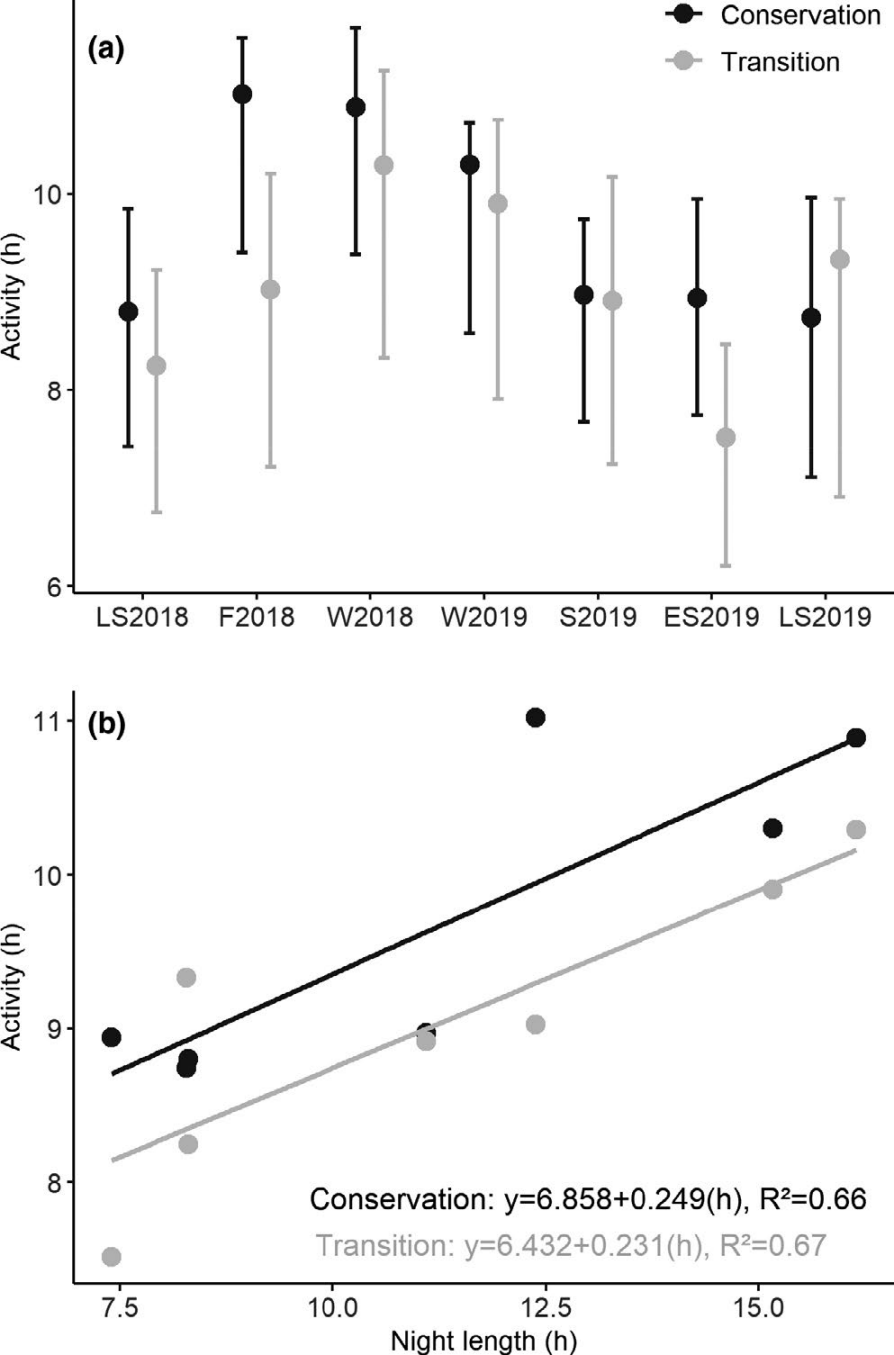
(a) Bimonthly (LS2018: July–August 2018; F2018: September–October 2018; W2018: November–December 2018; W2019: January–February 2019; S2019: March–April 2019; ES2019: May–June 2019; LS2019: July–August 2019) activity levels (estimated in hours active day^−1^) and associated 95% confidence intervals for wild boar (*Sus scrofa*) in the conservation and transition zones of the Schaalsee Biosphere Reserve, Germany; (b) depicts the relationship between wild boar activity (measured in hours day^−1^) and the night length for each bimonthly period

The analyses of our camera trap data indicated that the bimonthly diel activity distributions of the observed wild boar were predominantly characterized by nocturnal behavior. This was consistent throughout the bimonthly periods and in both the conservation and transition zones of the biosphere reserve (Figure 3). During most bimonthly periods, wild boar exhibited singular activity peaks during the first half or the middle of the night (fall 2018, winter 2018, winter 2019, and spring 2019). However, during the summer months (late summer 2018, early summer 2019, and late summer 2019), activity peaked in the first half of the night and the time before sunrise (Figure 3). The pairwise, time‐matched comparisons indicated that the diel activity patterns in the conservation and transition zones overlapped to a large degree (Table 1) and generally did not differ significantly between zones. However, during spring 2019 and early summer 2019, the daily distributions differed significantly between zones (Table 1). Although they were mostly nocturnal, the wild boar exhibited some diurnal activity (Figure 4), especially in the early morning and early evening (Figure 3). The proportion of diurnal activity was particularly noticeable during the summer periods (late summer 2018, early summer 2019, late summer 2019) and tended to be greater in the conservation zone than in the transition zone (Figure 4). The diel activity levels ranged from 7.5 to 11 h day^−1^. Over the course of the year, the bimonthly diel activity levels of wild boar fluctuated considerably in the conservation and transition zones (Figure 5). The seasonally matched diel activity level estimates tended to be slightly lower in the transition zone than in the conservation zone, yet these differences were significant during only the fall 2018 period (Table 2). Fall 2018 was also the period with the highest diel activity levels in the conservation zone (11 h day^−1^, 95% CI: 9.4–11.6), followed by only slightly lower values during the following winter periods (W2018: 10.9 h day^−1^, 95% CI: 9.4–11.7; W2019: 10.3 h day^−1^, 95% CI: 8.6–10.7). Similarly, diel activity levels in the transition zone were the highest during the winter seasons (2018: 10.3 h day^−1^, 95% CI: 8.3–11.3; 2019: 9.9 h day^−1^, 95% CI: 7.9–10.8) and the lowest in early summer 2019 (7.5 h day^−1^, 95% CI: 6.2–8.5) (Figure 5a). Overall, wild boar diel activity levels were slightly higher in the conservation zone than in the transition zone (Figure 5b), yet the pairwise comparisons revealed significant differences in diel activity levels during only the fall 2018 period (Table 2). The bimonthly diel activity level estimates were positively associated with night length, and the linear regression models explained a substantial amount of the observed variation in estimated diel activity levels (*R* ^2^ = 0.66 for the conservation zone; *R* ^2^ = 0.67 for the transition zone) (Figure 5b).

**TABLE 1:** Test statistics from randomization tests (1000 bootstrap iterations) of the probability that bimonthly (LS2018: July–August 2018; F2018: September–October 2018; W2018: November–December 2018; W2019: January–February 2019; S2019: March–April 2019; ES2019: May–June 2019; LS2019: July–August 2019) circular observations of wild boar in the conservation zone are from the same distribution as the circular observations of wild boar in the transition zone of the Schaalsee Biosphere Reserve, Germany

	Observed overlap	Mean null overlap	SE of null distribution	*p*‐value
LS2018	0.877	0.916	0.023	.061
F2018	0.877	0.917	0.025	.314
W2018	0.891	0.928	0.022	.054
W2019	0.903	0.928	0.022	.149
S2019	0.886	0.931	0.019	.019
ES2019	0.870	0.926	0.020	.007
LS2019	0.850	0.876	0.029	.172

**TABLE 2:** Test statistics from the Wald test for differences between bimonthly (LS2018: July–August 2018; F2018: September–October 2018; W2018: November‐December 2018; W2019: January–February 2019; S2019: March–April 2019; ES2019: May–June 2019; LS2019: July–August 2019) estimates of wild boar activity levels in the conservation zone and the transition zone of the Schaalsee Biosphere Reserve, Germany

	Difference	SE of the difference	W‐score	*p*‐value
LS2018	0.023	0.039	0.359	.549
F2018	0.083	0.040	4.230	.040
W2018	0.025	0.040	0.383	.536
W2019	0.017	0.038	0.185	.667
S2019	0.002	0.039	0.003	.954
ES2019	0.059	0.034	3.018	.082
LS2019	−0.025	0.045	0.302	.583

**FIGURE 6:**
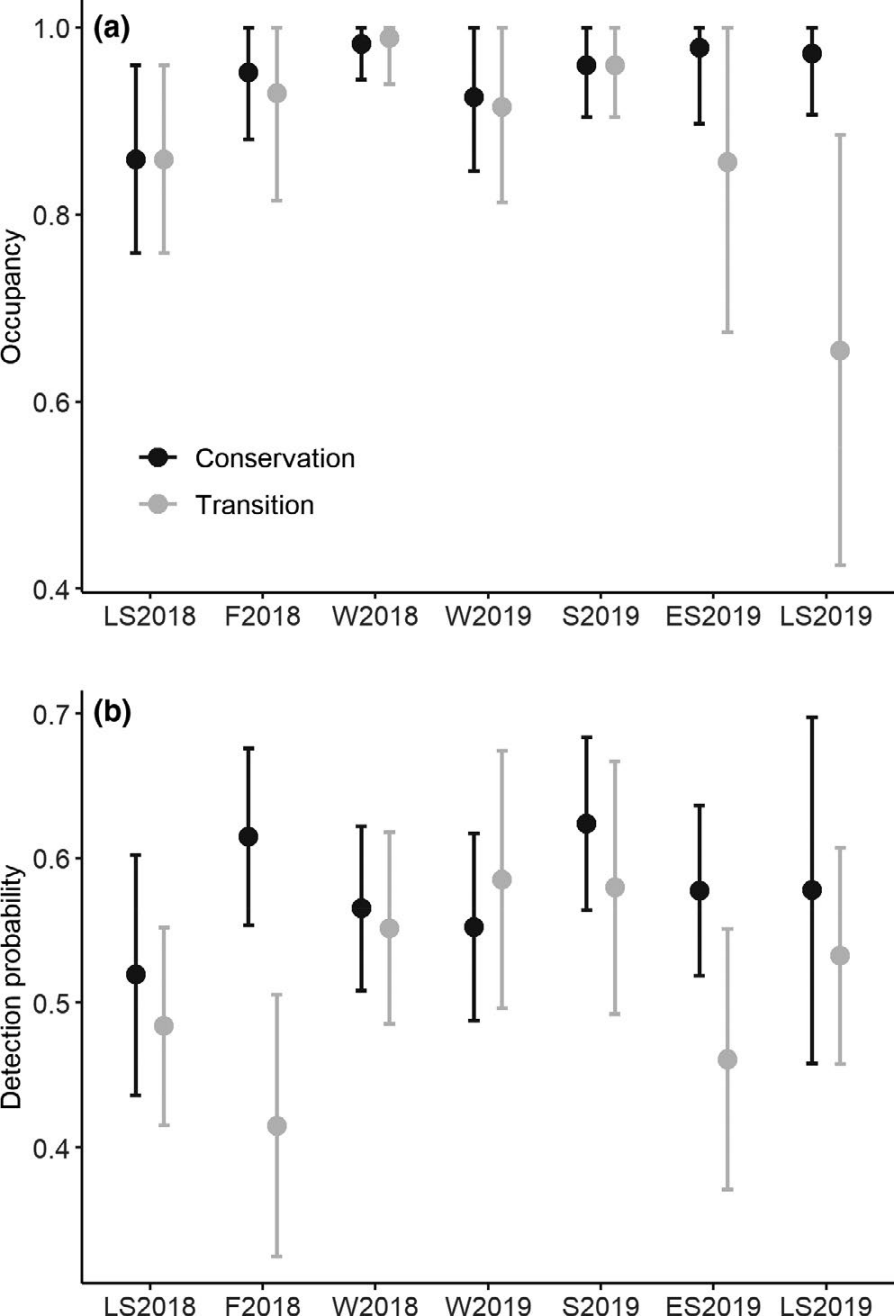
(a) Bimonthly (LS2018: July–August 2018; F2018: September–October 2018; W2018: November–December 2018; W2019: January–February 2019; S2019: March–April 2019; ES2019: May–June 2019; LS2019: July–August 2019) model‐averaged occupancy estimates and associated 95% confidence intervals for wild boar (*Sus scrofa*) in the conservation and transition zones of the Schaalsee Biosphere Reserve, Germany; (b) shows the model‐averaged detection probability and associated 95% confidence intervals for each bimonthly period and zone

Model selection often highlighted the intercept‐only models (LS2018, W2018, W2019, S2019) and models where detection probability was conditional on the zone (LS2018, F2018, W2018, W2019, S2019, and ES2019) as the best‐performing models. During four survey periods (F2018, S2019, ES2019, and LS2109), there was some support for the hypothesis that wild boar occupancy was different across zones (Table 3). Model‐averaged estimates indicate high wild boar occupancy in both zones of the biosphere reserve throughout the study period (Figure 6a), with model‐averaged point estimates ranging from 0.65 to 0.99. Across most (5/7) bimonthly periods, there was weak evidence for occupancy estimates of wild boar to differ between the zones of the reserve (Figure 6a). However, during the early and late summer period 2019, we found strong support for wild boar occupancy to be lower in the transition zone than in the conservation zone (Table 3; Figure 6a). The detection probabilities of wild boar were fairly constant across the bimonthly periods, with model‐averaged point estimates ranging between 0.42 and 0.62 (Figure 6b). Model selection generally provided support for slightly lower detection probabilities in the transition zones compared to the conservation zone, especially during the winter of 2018 and early summer of 2019 (Figure 6b; Table 3).

**TABLE 3:** Model selection table, showing key statistics of candidate models for estimating bimonthly (LS2018: July–August 2018; F2018: September–October 2018; W2018: November–December 2018; W2019: January–February 2019; S2019: March–April 2019; ES2019: May–June 2019; LS2019: July–August 2019) wild boar occupancy in the Schaalsee Biosphere Reserve, Germany

Model	*p* (Intercept)	*psi* (Intercept)	*p* (zone)	*psi* (zone)	df	logLik	AICc	ΔAICc	Weight
LS2018									
1	.02	1.81			2	−258.78	521.80	0.00	0.40
2	.14	1.80	+		3	−257.81	522.20	0.33	0.34
3	.02	1.66		+	3	−258.67	523.90	2.04	0.14
4	.15	1.64	+	+	4	−257.67	524.30	2.44	0.12
F2018									
2	.47	2.85	+		3	−258.58	523.70	0.00	0.68
4	.47	3.37	+	+	4	−258.16	525.30	1.55	0.31
1	.20	2.71			2	−264.99	534.30	10.55	0.00
3	.20	3.39		+	3	−264.40	535.40	11.64	0.00
W2018									
1	.24	4.25			2	−267.67	539.60	0.00	0.46
2	.32	4.13	+		3	−267.19	540.90	1.32	0.24
3	.24	3.61		+	3	−267.34	541.20	1.62	0.20
4	.33	3.55	+	+	4	−266.83	542.60	3.00	0.10
W2019									
1	.25	2.48			2	−240.27	484.80	0.00	0.38
2	.14	2.46	+		3	−239.25	485.00	0.22	0.34
3	.25	2.76		+	3	−240.05	486.60	1.82	0.15
4	.14	2.78	+	+	4	−238.98	486.90	2.07	0.13
S2019									
2	.56	3.17	+		3	−277.44	561.40	0.00	0.40
1	.44	3.16			2	−278.72	561.70	0.28	0.35
4	.56	3.44	+	+	4	−277.34	563.60	2.18	0.13
3	.44	3.44		+	3	−278.61	563.80	2.35	0.12
ES2019									
4	.30	7.60	+	+	4	−282.31	573.50	0.00	0.53
2	.33	2.54	+		3	−284.38	575.30	1.77	0.22
3	.16	8.98		+	3	−284.50	575.50	2.01	0.19
1	.18	2.53			2	−286.87	578.00	4.47	0.06
LS2019									
3	.26	3.59		+	3	−178.53	363.60	0.00	0.53
4	.38	3.50	+	+	4	−177.57	364.10	0.47	0.42
2	.39	1.89	+		3	−181.57	369.70	6.08	0.03
1	.26	1.79			2	−182.89	370.10	6.43	0.02

Note

We tested for candidate models in which detection probability (*p*) or occupancy (*psi*) are either constant (.) or conditional on the conservation zone (zone).

## 4 DISCUSSION

Most landscapes across the globe are now human‐dominated (Ellis et al., 2021). Understanding how wildlife alters its behavior in such landscapes is thus important for effectively managing the positive and negative impacts of wildlife (Ceausu et al., 2019; Neumann et al., 2021) and finding solutions to achieve coexistence between wildlife and people (König, Carter, et al., 2021; König et al., 2020). Here, we analyzed wild boar diel activity patterns across the different conservation zones of a biosphere reserve, which represent different levels of disturbance, using a systematic camera trap survey and occupancy modeling. Our findings highlight that during most of the study period, wild boar diel activity was not notably different between the zones with different levels of disturbance. Occupancy levels were generally high throughout the reserve, indicating that zoning had little influence on wild boar space use. Considering that wild boar numbers in Europe have been increasing strongly recently, resulting in growing conflicts with agriculture and conservation, our results highlight that wild boar population management should be aligned with other land management goals. More generally, our study shows that structuring a landscape into zones with different disturbance levels alone does not essentially influence the diel activity patterns of or space use by wild boar.

### 4.1 Wild boar nocturnality and human activity

Our results are well in line with studies on the diel activity patterns of wild boar subject to hunting pressure (Boitani et al., 1994; Brivio et al., 2017; van Doormaal et al., 2015; Johann, Handschuh, Linderoth, Dormann, et al., 2020; Keuling et al., 2008b; Ohashi et al., 2013; Podgorski et al., 2013; Russo et al., 1997), as our 14‐month‐long camera trap study suggests that the wild boar in the Schaalsee Biosphere Reserve in northern Germany were primarily nocturnal (Figures 3 and 4). Although the wild boar is often classified as nocturnal (Bennie et al., 2014), there is ample evidence that wild boars exhibit substantial diurnal activity and tend to be rather cathemeral if they are not subject to hunting (de Assis Morais et al., 2020; Johann, Handschuh, Linderoth, Dormann, et al., 2020; Podgorski et al., 2013). Shifts to nocturnal behavior in response to human disturbance have been observed in multiple species, and such shifts have been hypothesized to affect individual fitness, population dynamics, species interactions, and evolution (Gaynor et al., 2018). In wild boar, adjusting activity to times when humans are less active seems to be a fitness‐enhancing strategy, as indicated by the increasing wild boar populations across Europe (Carpio et al., 2020; Keuling et al., 2013, 2016; Massei et al., 2015).

### 4.2 Wild boar nocturnality and seasonality

Our analyses provided only partial support for the hypothesis that conservation zoning in a biosphere reserve has a marked effect on wild boar diel activity patterns. In five of the seven selected bimonthly periods, we did not detect significant differences in diel activity patterns between the conservation and transition zones (Table 1). However, during the summer seasons, the diurnal activity of wild boar was consistently greater in the conservation zone than in the transition zone (Figure 4); this result supports our first hypothesis. The apparent seasonal aspect of this effect could indicate that human disturbances in the different management zones differ seasonally (e.g., increased human activity in the agricultural fields in the transition zone) and that wild boars adaptively respond to such differences. In our study area, as well as in other study areas across Germany (Erdtmann & Keuling, 2020; Johann, Handschuh, Linderoth, Dormann, et al., 2020; Keuling et al., 2008b), wild boar spent more than half of the day resting (Figure 5). In our study, the active time that included movement, foraging, and social interactions was positively correlated with the night length (Figure 5b (compare also Keuling et al., 2008b)), indicating that wild boar diel activity is constrained to times when it is dark (Figures 3 and 4). Thus, the diurnal activity around dusk and dawn we found during the summer months (Figure 3) may merely be required to allow for sufficient foraging time during periods of the year when the nights are short (Keuling et al., 2008a, 2008b). The positive association between night length and diel activity level is consistent with the hypothesis that wild boar use the cover of the night for their activities (compare also Keuling et al. (2008b) in a neighboring study area), but our results showing high wild boar diel activity levels during the winter months are in contrast to the wild boar diel activity patterns observed in southern Germany. Based on data from accelerometers fitted to wild boars, Johann, Handschuh, Linderoth, Dormann, et al. (2020) found the highest wild boar diel activity during the summer months. Keuling et al. (2008b) also found the highest activity rates per hour in summer, while the total duration of activities per 24‐h cycle remained similar year‐round. We suspect that differences in environmental and weather conditions [e.g., there may have been more snow cover during the winter months in the study of Johann, Handschuh, Linderoth, Dormann, et al. (2020) than in our study] could explain such differences in seasonal diel activity levels (Lemel et al., 2003). Interestingly, Johann, Handschuh, Linderoth, Dormann, et al. (2020) found one activity peak around midnight, which is broadly in line with our results for the fall, winter, and spring periods (Figure 3). However, during the summer months in 2018 and 2019 (both summers were exceptionally warm), wild boar activity in our study area peaked just before sunrise (Figure 3). Because wild boar adjust their diel activity to the air temperature and avoid being active at temperatures above a region‐specific threshold (Figure 5 in Johann, Handschuh, Linderoth, Dormann, et al. (2020)), it is therefore plausible that wild boar are preferentially active during the comparatively cold times before sunrise during the summer months. In sum, our study suggests that conservation zoning affects the diel activity patterns and budgets of wild boar only during the summer months and only to a relatively small extent. Generally, wild boar diel activity in our study area was primarily restricted to the nighttime and may also have been mediated by the ambient temperature.

### 4.3 Do wild boar adjust their space use?

Our camera trap survey revealed high estimates of wild boar occupancy in both the transition and conservation zones of the biosphere reserve, suggesting that the species was widely distributed across the reserve. Except for the early and late summer of 2019 (when there was relatively strong support for occupancy to be lower in the transition zone than in the conservation zone), the occupancy estimates were similar across the zones of the biosphere reserve (Table 3; Figure 6). Thus, similar to our conclusion regarding the activity pattern hypothesis, our results only partially support the hypothesis that zoning affects space use by wild boar. The literature describes contrasting effects of seasonality on wild boar movement in Central Europe. Morelle and Lejeune (2015) suggested that wild boar populations in Belgium increase their range during the growing season, whereas Johann, Handschuh, Linderoth, Dormann, et al. (2020) found that GPS‐collared wild boar in southern Germany expanded their home range sizes during the winter months. Without more detailed information on the movement patterns of individual wild boars within our study area, it is difficult to hypothesize why wild boar occupancy tended to be lower in the transition zone during the summer months. More generally, however, our camera trap survey does not necessarily support the hypothesized seasonal shift in the distribution of wild boar (Keuling et al., 2009; Morelle & Lejeune, 2015) from forests (mostly in the conservation zone) during the nongrowing season to agricultural areas (mostly in the transition zone) during the growing season. Given the high occupancy estimates in both zones, it is plausible that the wild boar population in the Schaalsee Biosphere Reserve occurs at a locally high density. As resources are limited, habitat selection is likely density‐dependent (Avgar et al., 2020), making it hard to reveal habitat suitability and selection for generalists, such as wild boar (Morelle et al., 2015). Disentangling whether conservation zoning has relevant fitness consequences for wild boar would require a more detailed spatial analysis that outlines which areas are sources (i.e., mapping effective recruitment) and which areas are sinks (i.e., mapping mortality) for wild boar sounders (e.g., Mosser et al., 2009).

### 4.4 Implications for wild boar management

The main objectives of protected areas are to maintain ecosystems and their biodiversity, (Pullin et al., 2013), along with social and development goals, such as opportunities to experience and observe wildlife (Arbieu et al., 2017). With regard to conservation goals, wild boar rooting could have impacts on biodiversity (Cuevas et al., 2010, 2012). Likewise, socio‐economically wild boar could have both negative impacts (e.g., crop damages) and positive impacts (e.g., more wildlife experiences) (Ceausu et al., 2019; König, Ceaușu, et al., 2021). The zoning of protected areas is a potentially effective way to manage these trade‐offs in space, yet the transboundary effects of wild boar foraging outside protected areas and causing damage to farmland are major challenges (Amici et al., 2012; Geisser & Reyer, 2004; König et al., 2020; Schley et al., 2008). Our finding that zoning had little effect on wild boar diel activity and space use shows that this trade‐off is not effectively managed through zoning alone. We therefore suggest that there are multiple, nonmutually exclusive explanations for this finding. First, wild boars may not perceive strong differences between zones with respect to human influences, as they are hunted in both zones. Second, the current core zone in the Schaalsee Biosphere Reserve is patchily distributed and relatively small, which might explain the only slight differences we detected between zones. Finally, the apparently high boar densities require boars to use less suitable areas, and wild boars may use avoidance behavior at small spatio‐temporal scales to avoid interference with humans (Stillfried et al., 2017).

Wild boars can benefit from protected areas, as these provide refuges (Colomer et al., 2021). Although our study did not reveal strong effects of zoning on the spatio‐temporal behavior of wild boars, our results indicate that the animals intensively use the protected areas when moving through their agriculturally characterized habitat. For this reason, we assume that the core zones of the UNESCO Biosphere Reserve Schaalsee, which are small and patchily distributed, and provide important stepping stones for wild boars, ensuring the connectivity of habitats within the reserve. Given the substantial capacity and flexibility of wild boar reproduction (Frauendorf et al., 2016; Gamelon et al., 2017; Servanty et al., 2009; Vetter et al., 2020; Veylit et al., 2020), their numbers can grow rapidly, with possible repercussions for agriculture (Amici et al., 2012; Geisser & Reyer, 2004; Schley et al., 2008). Additionally, wild boar are susceptible hosts and potential vectors of economically relevant pathogens (Ruiz‐Fons, 2015; Vicente et al., 2002) and are themselves affected by them, such as in the case of the recent spread of African swine fever in Europe (Costard et al., 2009; Cwynar et al., 2019). While reducing the population densities of wild boar is a key control measure (O'Neill et al., 2020), their widespread use of the landscape, their high population densities, and their flexible and nocturnal behavior even in protected areas present challenges for population control. Thus, population management of wild boars should focus on areas neighboring protected areas. Beyond sport hunting as a means to control wild boar populations, authorities may consider additional measures (Keuling et al., 2016, Keuling et al., 2021), such as employing professional hunters equipped with night vision technology, or trapping of wild boars to effectively control the species (compare Gaskamp et al., 2021). Likewise, given the challenges and costs involved in controlling boar populations via hunting, the return of wolves, which feed on wild boars, should be seen as an opportunity to indirectly assist other management efforts to manage the space use of currently overabundant large ungulates (Rossa et al., 2021).

## CONFLICT OF INTEREST

None declared.

## AUTHOR CONTRIBUTIONS

**Henrik Reinke:** Conceptualization (lead); data curation (lead); formal analysis (equal); methodology (equal); validation (equal); writing–original draft (lead); writing–review and editing (equal). **Hannes J. König:** Conceptualization (equal); funding acquisition (lead); project administration (lead); resources (lead); supervision (lead); writing–original draft (equal); writing–review and editing (equal). **Oliver Keuling:** Conceptualization (supporting); supervision (supporting); writing–review and editing (supporting). **Tobias Kuemmerle:** Conceptualization (supporting); methodology (supporting); supervision (equal); writing–original draft (supporting); writing–review and editing (equal). **Christian Kiffner:** Formal analysis (lead); visualization (equal); writing–original draft (equal); writing–review and editing (equal).

## Data Availability

The wild boar camera trap data are available at dryad.org: doi:https://doi.org/10.5061/dryad.ngf1vhhvn
